# Combination of adenoviral virotherapy and temozolomide chemotherapy eradicates malignant glioma through autophagic and apoptotic cell death *in vivo*

**DOI:** 10.1038/sj.bjc.6604969

**Published:** 2009-03-10

**Authors:** I V Ulasov, A M Sonabend, S Nandi, A Khramtsov, Y Han, M S Lesniak

**Affiliations:** 1 The Brain Tumor Center, The University of Chicago, Chicago, IL 60637, USA; 2 Department of Pathology, The University of Chicago, Chicago, IL 60637, USA

**Keywords:** oncolytic adenovirus, brain tumour, glioma, temozolomide, apoptosis, autophagy

## Abstract

Conditionally replicative adenoviruses (CRAds) represent a novel treatment strategy for malignant glioma. Recent studies suggest that the cytopathic effect elicited by these vectors is mediated through autophagy, a form of programmed cell death. Likewise, temozolomide (TMZ), a chemotherapeutic agent used for the treatment of malignant gliomas, also triggers autophagic cell death. In this study, we examined the potential to combine the two treatments in the setting of experimental glioma. *In vitro*, pretreatment with TMZ followed by CRAd-Surivin-pk7 enhanced cytotoxicity against a panel of glioma cell lines. Western blot analysis showed increased expression of BAX and p53, decreased expression of BCL2 and elevated level of APG5. Treatment with TMZ followed by CRAd-Survivin-pk7 (CRAd-S-pk7) led to a significant over-expression of autophagy markers, acidic vesicular organelles and light-chain 3 (LC3). These results were further evaluated *in vivo*, in which 90% of the mice with intracranial tumours were long-term survivors (>100 days) after treatment with TMZ and CRAd-S-pk7 (*P*<0.01). Analysis of tumours *ex vivo* showed expression of both LC3 and cleaved Caspase-3, proving that both autophagy and apoptosis are responsible for cell death *in vivo*. These results suggest that combination of chemovirotherapy offers a powerful tool against malignant glioma and should be further explored in the clinical setting.

Malignant gliomas account for close to 50% of all CNS tumours and among these, high-grade gliomas like glioblastoma multiforme (GBM) are the most common (Annegers *et al*, 1981; Louis *et al*, 2001). Conventional care of patients bearing these tumours includes surgical debulking followed by radiotherapy and adjuvant chemotherapy (Lesniak *et al*, 2001). In spite of this treatment, the median survival of patients with GBM remains close to 1 year from the time of diagnosis (Stupp *et al*, 2005). Such poor outcome has led to the exploration of a wide variety of novel therapies, and over the last decade some of them have been incorporated as a standard of care for patients with this disease. This is the case for temozolomide (TMZ), an alkylating chemotherapeutic agent that is available for the treatment of primary and recurrent high-grade gliomas, including anaplastic astrocytomas. Temozolomide became available after clinical trials showed its therapeutic benefit in these tumours. Specifically, a phase III randomized controlled clinical trial evaluated the role of TMZ in combination with radiotherapy for the treatment of patients with GBM (Stupp *et al*, 2005). In this study, radiotherapy and TMZ led to a median survival of 14.6 months compared with a median survival of 12.1 months for those patients treated with radiotherapy alone. The 2-year survival rate was 26.5% with radiotherapy and TMZ and 10.4% with radiotherapy alone.

Whereas the efficacy of TMZ is encouraging, additional prolongation of survival remains a challenge, and thus alternative therapeutic strategies are being explored. Some of these include immunotherapy, stem cell therapy, local chemotherapy and radiotherapy (Lesniak *et al*, 2001; Ehtesham *et al*, 2005; Lesniak, 2005; Kushen *et al*, 2007). Conditionally replicative adenoviruses (CRAds) represent a novel treatment strategy as well (Sonabend *et al*, 2006). Conditionally replicative adenovirus is a genetically modified adenovirus that preferentially replicates in and kills tumour cells, and ideally is unable to replicate in normal cells. As such, culmination of every viral reproductive cycle leads to cell destruction and release of new viral particles. This progeny is once more able to infect neighbouring neoplastic cells, further enhancing oncolysis.

Recent studies suggest that the cytopathic effect elicited by some CRAd in gliomas is mediated through autophagy (Ito *et al*, 2006; Jiang *et al*, 2007), a form of programmed cell death. Likewise, TMZ also triggers glioma cell death through autophagy (Kanzawa *et al*, 2004). Therefore, we hypothesised that co-treatment of glioma cells with TMZ and CRAd might lead to an enhanced therapeutic effect. In this context, we tested the efficacy of this chemotherapy–virotherapy combination with TMZ and CRAd-Survivin-pk7 (CRAd-S-pk7), a novel oncolytic adenoviral vector that utilises the survivin promoter to drive E1A expression and binds to heparan sulphate proteoglycans expressed on malignant glioma (Ulasov *et al*, 2007; Zheng *et al*, 2007; Nandi *et al*, 2008). We evaluated this combination in different regimes and tested its efficacy *in vitro* and *in vivo.* Furthermore, we explored the possible mechanisms, by which this combination might elicit therapeutic efficacy, highlighting the differences observed *in vitro* to those seen *in vivo*.

## Materials and methods

### Cell lines

The human glioma U373MG and U87MG were purchased from ATCC and propagated in monolayer culture in RPMI 1640 supplemented with 10% foetal calf serum. Human glioma cells lines Kings and No.10 (Japan Tissue Bank, Tokyo, Japan) were maintained in RPMI 1640 supplemented with 10% FBS (HyClone, Logan, UT, USA), 2 mmol l^−1^ of L-glutamine, 100 *μ*mol l^−1^ of MEM nonessential amino acids (Invitrogen Corporation, Carlsbad, CA, USA) and 1 mM l^−1^ of sodium pyruvate (Sigma-Aldrich, St Louis, MO, USA).

### Chemotherapeutic agents and adenoviral vectors

Temozolomide was kindly supplied by the Schering-Plough Research Institute (Kenilworth, NJ, USA) and was dissolved in DMSO (Sigma Chemical Co., St Louis, MO, USA) to produce a 100 mM stock solution for *in vitro* experiments. A total of 100 mM stock solution was dissolved into 2% serum containing media (for *in vitro* experiments) to obtain 1, 5, 10, 25, 50, 75, 100, 250 and 500 *μ*M solutions. For *in vivo* experiments, stock solution was diluted in serum-free media to make 3.2 mg ml^−1^ solution before injection. A dose of 10 mg kg^−1^ body weight was used, which corresponded to a dose of 25 mg kg^−1^ m^−2^ in humans.

The 3-MA inhibitor was purchased from Sigma (St Louis, MO, USA) and Bafilomycin A1 (BAF-A1) was purchased from EMD Biosciences (San Diego, CA, USA).

The CRAd-S-pk7 adenoviral vector has been described earlier by our group (Ulasov *et al*, 2007). Briefly, the survivin-controlled E1 expression cassette was placed in the native E1 region of the Ad genome to avoid nonspecific viral replication. Recombinant adenoviruses were created on the basis of homologous recombination in 911 cells between a shuttle vector – pScs/PA/S, which carries a human survivin promoter and a pVK700-based adenoviral wild-type five backbone containing polylysine modification in the fibre knob (CRAd-S-pk7). Conditionally replicative adenovirus was selected from a single plaque on 911 cells, expanded in A549, and then purified by double CsCl gradient ultracentrifugation (Graham *et al*, 1977).

### Analysis of adenovirus-mediated toxicity in combination with a chemotherapeutic agent

For determination of the cytotoxic effect mediated by our treatment, cells were grown in 96-well plates (1 × 10^4^ cells per well) for 24 h and subsequently incubated with CRAd-S-pk7 (100 vp per cell), TMZ (100 *μ*M) or both for 24 h. Toxicity was measured by using CytoTox-ONE Homogeneous Membrane Integrity Assay kit (Promega, Madison, WI, USA). All experiments were performed twice with eight wells per condition.

For inhibitory studies, U87MG cells were grown in a 96-well plate (1 × 10^4^ cells per well) for 24 h in 100 *μ*l volume. After overnight incubation, fresh media supplemented with either 3-MA (Sigma) (10 mM) (Ropolo *et al*, 2007) or BAF-A1, (EMD Biosciences) (10 nM) (Kanzawa *et al*, 2004) was added for further incubation. Next day, the media containing the drug was removed and the cells were treated with CRAd-S-pk7 (100 vp per cell) or TMZ (100 *μ*M, both for single and co-treated groups) for 24 h. After 24 h, the group consisting of both treatments received additional dose of CRAd-S-pk7 (100 vp per cell), and the incubation continued for the next 96 h. Toxicity was measured by using CytoTox-ONE Homogeneous Membrane Integrity Assay kit (Promega). All experiments were performed twice and expressed as average toxicity of six wells per drug.

### SYBR-Green real-time quantitative PCR analysis of gene expression

Total DNA was isolated using DNA tissue kit (Qiagen, Valencia, CA, USA) and quantitative PCR analysis was performed on a OPTICON 2-detection system (Bio-Rad Laboratories, Hercules, CA, USA) using a SYBR-green PCR master mix (Applied Biosystems, Foster City, CA, USA), and specific primers for E1A (forward: 5′-AACCAGTTGCCGTGAGAGTTG; reverse: 5′-CTCGTTAAGCAAGTCCTCGATACAT) and the house-keeping gene *β*-actin (forward: 5′-TTTATCCGCCTCCATCCA; reverse: 5′-CAAACGACGAGCGTGACA). Data were analysed with Opticon 2 Software (Bio-Rad Laboratories) and adenoviral genome titre in copy numbers per nanogram cellular genomic DNAs were determined. All experiments were performed in triplicates.

### Western blot analysis

1.0 × 10^6^ U87MG cells were washed and pelleted at 0 and 5 days after CRAd-S-pk7 (100 vp), TMZ (100 *μ*M) or combination treatments and lysed using M-PER lysis buffer (Pierce, Rockford, IL, USA) containing Protease and Phosphatase inhibitors (Pierce). The protein extracted was quantified using a Bradford assay (Bio-Rad Laboratories). A total of 50 *μ*g of each sample was heated at 95°C for 10 min and loaded into 4–20% gel (Bio-Rad Laboratories). Samples were resolved on the gel at 150 V for 60–90 min and transferred to PVDF membranes at 15 V for 1 h using a semi-dry transfer apparatus (Bio-Rad Laboratories). Membranes were blocked in 5% non-fat dry milk for 2 h, incubated with primary antibodies for 2–16 h, washed with TBS containing 0.05% Triton-X 100 (TBST) followed by an incubation of 30 min to 1 h in appropriate secondary antibody conjugated with HRP (Santa Cruz Biotechnology, Santa Cruz, CA, USA). After final washing with TBST, the membranes were developed using SuperSignal West Pico Chemiluminescent reagent (Pierce) and exposed to X-ray films (Pierce). Antibodies used were: Adenovirus type 5 E1A ab-1 (M58, LabVision, Fremont, CA, USA), *α*-p53 ab-6 (DO-1, LabVision), *α*-p21^WAF1/Cip1^ (SX118, Dako, Carpinteria, CA, USA), *α-*Cdc20 (AR12, Millipore, Billerica, MA, USA), *α*-NIP3 (Ana40, Santa Cruz Biotechnology), *α*-BCL-XL (7B2.5, Chemicon, Billerica, MA, USA), *α*-BIK (#4592, Cell Signaling, Danvers, MA, USA), *α*-BAX (#2772, Cell Signaling), *α*-NOXA (Santa Cruz Biotechnology), *α*-BAD (#9292, Cell Signaling) *α*-PUMA (#4976, Cell Signaling), *α*-BCL2 (#2876, Cell Signaling), *α*-Caspase-3, which recognises both cleaved and uncleaved Caspase-3 (Cell Signaling), *α*-BID (#550365, BD Biosciences, San Diego, CA, USA), *α*-APG5 (#FL275, Santa Cruz Biotechnology) and *α*-beclin (H-300, Santa Cruz Biotechnology). Equal loading was confirmed by probing with pan-actin monoclonal antibody (AC-15, ABCAM, Cambridge, MA, USA).

### Flow cytometric analysis of glioma cells

Fifty thousand U87MG cells were plated 24 h before treatment. Next day, cells were treated with CRAd-S-pk7 (100 vp per cell) or TMZ (100 *μ*M) or a combination of CRAd-S-pk7 (100 vp per cell) and TMZ (100 *μ*M) for 24 h. After 24 h, to obtain combination of both drugs, cells from co-treated groups received either mock, CRAd-S-pk7 (100 vp per cell) or TMZ (100 *μ*M) treatments for next 96 h. All cells from the wells, floating and attached, were collected, washed and divided into three portions: one portion was fixed with 4% paraformaldehyde in 0.1 M phosphate buffer, washed and permeabilised with Triton X-100 for 5 min followed by labelling with rabbit *α*-LC3B (NB600-1384, Novus Biologicals, Littleton, CO, USA) for 2 h at room temperature. After washing thrice with phosphate-buffered saline (PBS), cell suspension was stained at 4°C with FITC-conjugated goat *α*-rabbit (sc2012, Santa Cruz Biotechnology) or IgG_1_ isotype control antibody (BD Pharmingen, San Diego, CA, USA) for 60 min. Cells were again washed thrice with PBS, resuspended into PBS and amounts of LC3B-positive cells were analysed by flow cytometry using a FACSCalibur (Becton Dickinson, San Jose, CA, USA). The FlowJo program (Becton Dickinson) was used for data analysis. The second portion was labelled with acridine orange (AO) solution (1 *μ*g ml^−1^, Sigma) for 15 min and subjected to flow cytometry for detection of autophagosomes.

### Annexin V staining, mitochondrial membrane potential assay and cell cycle analysis

Apoptosis was quantified using the Annexin V-PE apoptosis kit (BD Biosciences) according to the instructions of the manufacturer. Briefly, 2 × 10^5^ U87MG cells glioma cells were seeded in a 6-well plate 24 h before treatment. Next day, cells were treated with CRAd-S-pk7 (100 vp per cell) or TMZ (100 *μ*M) or combination of CRAd-S-pk7 (100 vp per cell) and TMZ (100 *μ*M) for 96 h, at which point cells were collected for staining. Staining for mitochondrial membrane potential was done using DiIC staining (Invitrogen Corporation) from the same set of samples as per manufacturer's recommendation. Samples were analysed using a FACScan (Becton Dickinson, Mountain View, CA, USA) Data was analysed with CellQuest software (Becton Dickinson).

To evaluate the distribution of treated cells on the basis of cell cycle properties, treated cells were washed twice with ice-cold PBS, collected by centrifugation and fixed in 70% (v/v) ethanol at 4°C for 2 h. After fixation, the cells were treated with 1 ml propidium iodide staining buffer (0.1% Triton X-100, 100 *μ*g ml^−1^ RNase A, 80 *μ*g ml^−1^ propidium iodide in PBS) at 37°C for 30 min. Cells were detected using a cytofluorometer, and analysed by FACScan and CellQuest program (Becton Dickinson). The experiment was performed twice in duplicates, and the average results from one experiment are presented in Table 1.

### *In vivo* human tumour model and histopathological study

All animal experimental protocols were approved by the Institutional Animal Care Committee of the University of Chicago. U87MG cells (0.3 × 10^6^ cells per mouse) were injected intracranially into the right lobe of 5- to 6-week-old female BALB/c *nu/nu* mice (*n*=13 per group). Four days after injection of tumour cells, few mice were killed to check for tumour growth. After the tumour was confirmed, the mice were randomized and treated with five consecutive intraperitoneal (i.p.) injections of TMZ (70 or 10 mg kg^−1^ body weight in 100 *μ*l) along with 5 *μ*l solution containing either AdWT or CRAd-S-pk7 at a dose of 3 × 10^9^ vp per mouse injected (one or two injections). The health of each group was carefully monitored and symptom-free survival time was noted. Sick animals were killed to avoid pain and suffering. One animal from each group was killed every 15 days, unless they needed to be killed due to deteriorating health to examine Intracranial (i.c.) tumour and analyse brain tissue. Brain tissues were fixed in 10% formalin, embedded in paraffin, and then cut into 5 *μ*m thick sections or used for tissue microarrays. DNA was isolated from 5 mm thick tissue section and processed for detection of adenoviral expressions by quantitative PCR using E1A primers and normalised to GAPDH.

For tissue microarray analysis, the paraffin blocks from five different groups: (mock; CRAd-S-pk7; TMZ (10 mg kg^−1^ per day); CRAd-S-pk7 and TMZ (10 mg kg^−1^ per day) were constructed as described by (Kononen *et al*, 1998). Sections stained with hematoxylin and eosin (H&E) were used to morphologically localise representative tumour regions within the blocks (5 per condition/per treatment). A tissue array instrument (Beecher Instruments, Silver Spring, MD, USA) was used to create holes in a recipient paraffin block and to acquire tissue cores from the donor block by a 1.0mm thin-walled needle held in an X–Y precision guide. The cylindrical samples were retrieved from the selected tumour regions in the tissue blocks and transferred directly into the recipient blocks with defined array coordinates. After the construction of the array block, multiple 5 *μ*m thick sections were deparaffinised and rehydrated using xylene and ethanol and then transferred to the 0.02% Triton for permeabilisation. Slides in citrate buffer (pH 6.0) were heated in the steamer for 30 min. After cooling for 30 min and a three 5 min wash in PBS, the slides were incubated in 3% BSA for 30 min. The slides were successively transferred to 3% H_2_O_2_ for 10 min and then incubated overnight with either rabbit monoclonal *α*-Ki-67 (M7187, Dako), *α*-LC3 (Novus Biologicals) or *α*-Caspase-3, which recognises cleaved Caspase-3 (Cell Signaling), after processing by EnVision system (Dako) according to the manufacturer's instructions. Negative control slides were processed in an identical fashion to that above, with the substitution of 1% BSA in PBS for the primary antiserum. Photomicrographs were taken from one mouse sample on a given time point (day) as representative image of whole group using a Leica DMLS microscope coupled to a digital camera (Photometrics SNAP, Roper Scientific, Inc., Glenwood, IL, USA).

### Quantitative analysis

To quantify cells positively stained for Ki-67, light-chain 3 (LC3) and cleaved Caspase-3, an Aperio ImageScope V8.0.39.1059 software (Aperio Technologies Inc., Vista, CA, USA) was used to assess the quantity and morphology of stained cells from TMA sections. Randomly chosen fields were captured by light microscope at × 20 magnification. Acquired images were analysed using ‘positive pixel count 2004-08-11’ algorithm as percent of positive cells in total tumour cell population per image.

### Statistical analysis

Comparison of toxicities was made by using a two-sided Student's *t*-test. Kaplan–Meier survival curves of animals treated with CRAd-S-pk7, TMZ or combination were estimated. The survival curves were compared based on log-rank test. *P*<0.05 was considered statistically significant.

To evaluate additive effect from both TMZ and CRAd-S-pk7, we defined the toxicity mediated by TMZ as P1 and toxicity mediated by CRAd-S-pk7 as P2. Assuming that combination of toxicity is iso-additive, the expected toxicity (*P*_e_) will be *P*_e_=P1+(1−P1) × P2. If the observed toxicity in combination condition is <*P*_e_, both drugs induce sub-additive effect; if the observed toxicity in combination condition is >*P*_e_, both drugs induce supra-additive effect.

Owing to the difference in staining between slices obtained from animals of same group, we first expressed the data in a logarithmic (base 10) scale. The pattern of staining (after log transformation) as a function of days after treatment was not linear; therefore, polynomial regression models were used and the results indicated that a quadratic polynomial model would be sufficient to fit the data ((log_E_)=*β*_0_+*β*_1_day+*β*2day^2^, *R*^2^∼0.57). To compare the fitted curves among the conditions (mock, TMZ, CRAd-S-pk7 or combination therapy), a second order polynomial regression model with interaction between the condition and day was fitted: Log(Lg_E_)=*β*_0_+*β*_1_day+*β*_2_*G*+*β*_3_day^2^+*β*_4_day^*^G+*β*_5_day^2^^*^G+e, where *G* represents the conditions (group effect), day^*^G and day^2^^*^G represent the interaction terms, and e is the error term. A test for the null hypothesis *β*_2_=0, *β*_4_=0 and *β*_5_=0 was used to indicate whether there was difference in the fitted curves between conditions.

## Results

### Combination treatment of TMZ and CRAd-S-pk7 exhibits additive effect in glioma cell lines

**Figure 1:**
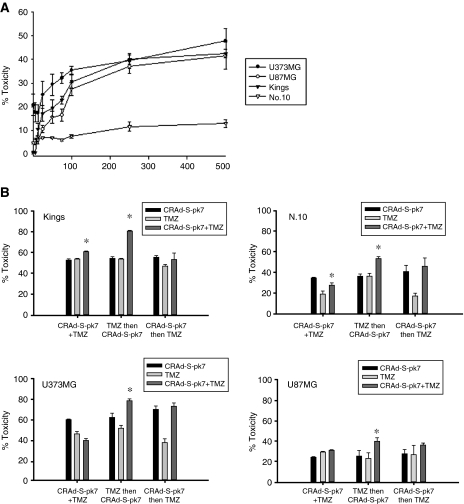
Temozolomide alone or in combination with CRAd-S-pk7 induces cell death that is additive in effect. (**A**) Growth-inhibitory effect of TMZ on human glioma cells. Kings, No.10, U87MG and U373MG cells were seeded at 10^4^ cells per well in 96-well plate and incubated overnight at 37°C. After exposure to TMZ (1, 5, 10, 50, 100, 250 and 500 *μ*M) for 120 h, the cells were subjected to cytotoxicity assay by detecting LDH release. Percentage of dead cells from two independent experiments was plotted for a dose-response curve. The four curves represent different sensitivity of human gliomas to TMZ treatments. (**B**) Comparison of the toxicity mediated by TMZ in the presence or absence of oncolytic virus. Cells were treated only with TMZ or CRAd-S-pk7; pretreated with TMZ and then infected with CRAd-S-pk7; or infected with CRAd-S-pk7 first and then treated with TMZ at doses indicated earlier. ^*^P<0.05 *vs* CRAd-S-pk7 or TMZ.

To investigate if the combination of CRAd-S-pk7 and TMZ leads to an enhanced cytotoxic effect in glioma, we first assessed the cytotoxicity of TMZ at different concentrations in U87MG, U373MG, Kings and No. 10 human glioma cells. Plated cells were exposed to TMZ at a concentration of 1, 5, 10, 25, 50, 75, 100, 250 and 500 *μ*M. After 5 days of incubation, the cytotoxicity was determined by a cell integrity assay (Figure 1A). In the case of U87MG, U373MG, and Kings cells, TMZ in the range of 0–100 *μ*M elicited cytotoxicity in a dose-response manner. Consistently, published data from patients involved in TMZ clinical trials reported similar plasma TMZ concentrations (Newlands *et al*, 1992; Brada *et al*, 1999). This effect did not increase in the same ratio with concentrations higher than 100 *μ*M of the chemotherapeutic agent. In fact, at higher doses, we observed a plateau. On the other hand, the No. 10 cell line remained relatively resistant to TMZ in comparison with other cell lines (*P*<0.05). On the basis of these results, a concentration of 100 *μ*M TMZ was used for further *in vitro* experiments.

To explore the possibility of additive cytotoxicity derived from co-treatment of glioma cells by CRAd-S-pk7 and TMZ, these two agents were combined in three alternative treatment sequences. The therapeutic schedules consisted of simultaneous incubation with CRAd-S-pk7 and TMZ, incubation with CRAd-S-pk7 followed by treatment with TMZ, or initial TMZ treatment followed by incubation with CRAd-S-pk7. We used CRAd-S-pk7 at a concentration of 100 vp based on therapeutic cytotoxicity observed in one of our earlier studies (Ulasov *et al*, 2007). The additive effect was defined as a significant increase in cytotoxicity of CRAd-S-pk7 and TMZ in comparison with therapy with either CRAd-S-pk7 or TMZ alone. With respect to cytotoxicity, treatment with CRAd-S-pk7 followed by TMZ showed no significant increase in cell death (Figure 1B). Similarly, simultaneous treatment with CRAd-S-pk7 and TMZ only led to enhancement of cell killing in Kings cell line (*P*<0.05). However, and of most importance, TMZ treatment followed by CRAd infection elicited an additive cytotoxic effect in all glioma cell lines evaluated (*P*<0.05) (Figure 1B). This effect was much higher than the effect mediated by TMZ or CRAd-S-pk7 alone.

### The additive effect observed by TMZ and CRAd-S-pk7 is mediated by autophagy *in vitro*

**Figure 2:**
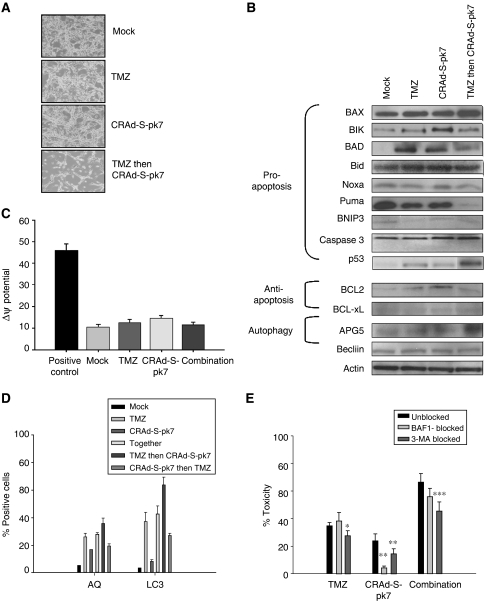
Induction of autophagy in U87MG cells treated with TMZ followed by CRAd-S-pk7 infection. Effect of combined treatment of U87MG cells was detected by (**A**) light microscopy, (**B**) western blot, (**C**) membrane potential, (**D**) flow cytometry and (**E**) LDH toxicity. (**A**) Decrease in cell density and morphological changes associated with treatment with TMZ followed by CRAd-S-pk7. (**B**) Modulation of pro-apoptotic, anti-apoptotic and autophagic proteins in response to treatment with TMZ (100 *μ*M), CRAd-S-pk7 (100 vp per cell) or combination (TMZ and CRAd-S-pk7) as determined by Western blot analysis. Pretreatment of U87MG cells with TMZ followed by CRAd-S-pk7 infection showed over-expression of p53, Bax and APG5 proteins and downregulation of Puma, Noxa, BNIP3 and BCL-2 proteins. There was no evidence of cleaved Caspase-3. (**C**) To show that mitochondrial pathway is not activated, we measured mitochondrial potential changes. TMZ, CRAd-S-pk7 and combination group did not induce significant changes in Δ*ψ*. (**D**) Autophagy was determined by staining with acridine orange (AO) and *α*-LC3B antibody followed by flow cytometry analysis. Experiment was performed in triplicates and the mean of two independent experiments is shown here. (**E**) Effect of Bafilomycin A1 (BAF-A1) and 3-MA treatments on co-treatment induced toxicity. Cells were pretreated with 3-MA, BAF-A1 or vehicle control for 12 h before exposure to TMZ, CRAd-S-pk7 or TMZ followed by CRAd-S-pk7. Figure summarises data from two independent experiments each having six replicates per condition. (^*^), (^**^) and (^***^) *P*<0.05. (^*^), (^**^) and (^***^) *P*-value determined by comparing mock *vs* TMZ alone, CRAd-S-pk7 alone or combination TMZ then CRAd-S-pk7, respectively.

On the basis of the finding that pretreatment with TMZ followed by CRAd-S-pk7 (combination therapy) leads to an enhanced cytotoxic effect in all cell lines tested, we investigated the mechanism behind this phenomenon in the U87MG cell line. Consistent with the cell integrity assay (Figure 1), treatment with TMZ followed by CRAd-S-pk7 led to a cytopathic effect characterised by a decrease in cell density and morphological changes (Figure 2A). To assess the possibility of cell death by activation of apoptosis, we evaluated the expression of proteins BAX, BIK, BAD, p53 and Caspase-3 (both cleaved and uncleaved), among other pro-apoptotic proteins, which have been implicated in the induction of apoptosis by adenovirus (Verma *et al*, 2001; Nahle *et al*, 2002; Zhang *et al*, 2003; Subramanian *et al*, 2007), as well as anti-apoptotic proteins BCL-2 and BCL-X_L_ (Shimazu *et al*, 2007) (Figure 2B). It is interesting to note that the expression pattern of some of these proteins points towards a pro-apoptotic stage triggered by treatment with TMZ followed by CRAd-S-pk7. This is suggested by the increase in expression of p53 and BAX, and a decrease in BCL-2 expression. On the other hand, the fact that the expression of BAD and PUMA was decreased, the lack of change in expression of BID and NOXA, and the absence of Caspase-3 cleavage along with absence of mitochondrial depolarisation (Figure 2C) do not support the hypothesis of classical apoptosis as the definitive mechanism for the observed cytotoxic effect elicited by this TMZ and CRAd-S-pk7 combination.

Whereas TMZ (Kanzawa *et al*, 2004) and some CRAd (Ito *et al*, 2006; Jiang *et al*, 2007) have been described to elicit cytotoxicity in glioma cells through autophagy *in vitro*, we tested this hypothesis in the context of TMZ and CRAd-S-pk7 combination therapy. APG 5 was found upregulated in the co-treated group in comparison with others (Figure 2B). To explore the possible induction of autophagy, we quantified the presence of acidic vesicular organelles (AVO) that are characteristic of this process and can be detected by flow cytometry with AO staining (Paglin *et al*, 2001; Daido *et al*, 2004; Kanzawa *et al*, 2004). In addition, we determined the expression of the microtubule-associated protein 1 LC3, a protein that plays a role in autophagy, as it is implicated in the formation of the autophagosomes (Kabeya *et al*, 2000; Mizushima *et al*, 2001; Munafo and Colombo, 2001). Treatment with TMZ followed by CRAd-S-pk7 led to a significant increase of AVO (*P*<0.05) and LC3 expression (*P*<0.05) (Figure 2D) in comparison with TMZ or CRAd-S-pk7 treatment alone 5 days after the initiation of treatment. These findings suggest the enhancement of autophagy induction when TMZ and CRAd-S-pk7 are combined. The decrease in BCL-2 expression observed by TMZ and CRAd-S-pk7 combination in comparison with TMZ or CRAd-S-pk7 alone (Figure 2B) could also indicate the presence of autophagy. This is suggested by the fact that besides its anti-apoptotic property, BCL-2 seems to inhibit autophagy by direct interaction with Beclin 1 (Saeki *et al*, 2000; Cardenas-Aguayo Mdel *et al*, 2003; Pattingre *et al*, 2005; Pattingre and Levine, 2006) and therefore, its downregulation could trigger the latter process.

The studies describing autophagic death of glioma cells secondary to TMZ or CRAd showed that such cytotoxicity is decreased by blocking autophagy (Kanzawa *et al*, 2004; Ito *et al*, 2006). To investigate whether the toxicity elicited by this treatment combination is affected by the blocking of autophagy, we assessed the toxicity elicited by TMZ, CRAd-S-pk7 or the combination in the presence of BAF-A1 or 3-MA, two agents that interfere with autophagy by preventing the fusion between autophagosomes and lysosomes (Yamamoto *et al*, 1998). Of note, treatment of cells with BAF-A1 leads to inhibition of maturation of autophagosome, whereas 3-MA inhibits autophagy at the formation of double-membrane structure when portion of the cytosol is sequesters from the rest of cytoplasm (Klionsky and Ohsumi, 1999; Ravikumar *et al*, 2002). In our inhibition experiment, the toxicity elicited by TMZ was similar in the presence of BAF-A1 (∼5% increase, *P*>0.05) and decreased in the presence of 3-MA (Figure 2E). On the other hand, cell death secondary to CRAd-S-pk7 was significantly diminished by the presence of BAF-A1 and 3-MA (*P*<0.05) (Figure 2E). Most importantly, the treatment combination of TMZ followed by CRAd-S-pk7 elicited a cytotoxic effect that was significantly impaired by the presence of 3-MA, suggesting that this cell death is secondary to autophagy (*P*<0.05) (Figure 2D).

### Role of mitotic catastrophe in TMZ and CRAd-S-pk7 combination-induced cell death

**Figure 3:**
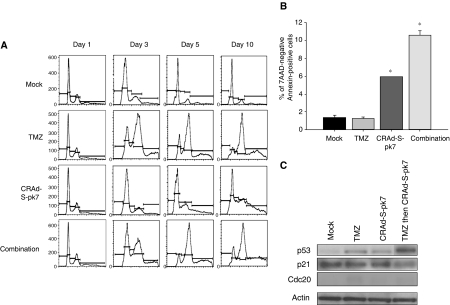
Mitotic catastrophe does not contribute to drug-induced toxicity and cell cycle arrest. U87MG cells were treated with either TMZ (100 *μ*M), CRAd-S-pk7 (100 vp per cell) or combination (TMZ and CRAd-S-pk7). Treated cells were harvested either on day 1, 3, 5 and 10 and subjected to (**A**) cell cycle distribution analysis, (**B**) Annexin V/7AAD assay, or (**C**) western blotting with antibodies recognising Cdc20 protein.

We investigated whether aberrant mitosis has an impact on TMZ- and TMZ and CRAd-S-pk7 combination-induced cell death. In cells, mitotic catastrophe (MC) is mainly associated with deficiency in cell cycle check points (Castedo *et al*, 2004; Thirthagiri *et al*, 2007; de Bruin and Medema, 2008; Wang *et al*, 2008). Cell cycle analysis (Figure 3A and Table 1) revealed that TMZ and combination-treated groups were arrested in G2/M phase (50.1% at day 10 compared with 22.6% at day 1 for TMZ and 38.4% at day 10 compared with 24.1% at day 1 in combination group), whereas CRAd-S-pk7 infection of U87MG cells led to G1 cell cycle arrest (49% at day 10 compared with 23.4% at day 1).

It is interesting to note that these G1 and G2/M arrests significantly increased the amount of polyploid cells. Thus, for TMZ, virus and combination treatment groups, we observed that 32.4, 30.85 and 40.5% cells, respectively, were polyploid at day 10. However, our results also indicate that both agents provide cell cycle arrest without significantly raising the sub-G1 population, thereby confirming the absence of DNA degradation and an absence of MC. Furthermore, to characterise cell death we performed AnnexinV/7AAD staining. As shown in the Figure 3B, TMZ treatment did not induce any changes in Annexin V staining. In comparison, cells treated with CRAd-S-pk7, and the combination treatment group showed an increase in cells positive for Annexin V staining with a concurrent absence of 7AAD staining, indicating early apoptosis (Figure 3B).

Finally, aberrant mitosis might be the result of defects in spindle (mitotic) checkpoints. As mitotic progression is associated with the interaction of Cdc20 with anaphase promoting complex resulting in its activation, we examined the expression of Cdc20 in mock, TMZ, CRAd-s-pk7 or combination-treated cells. As seen in Figure 3C, there was no evidence of elevated Cdc20 in any of the treatment groups, further excluding MC as a mechanism responsible for cell death in these treatment strategies.

### TMZ and CRAd-S-pk7 combination leads to therapeutic additive effect with an increase in survival of mice bearing intracranial glioma xenografts

**Figure 4:**
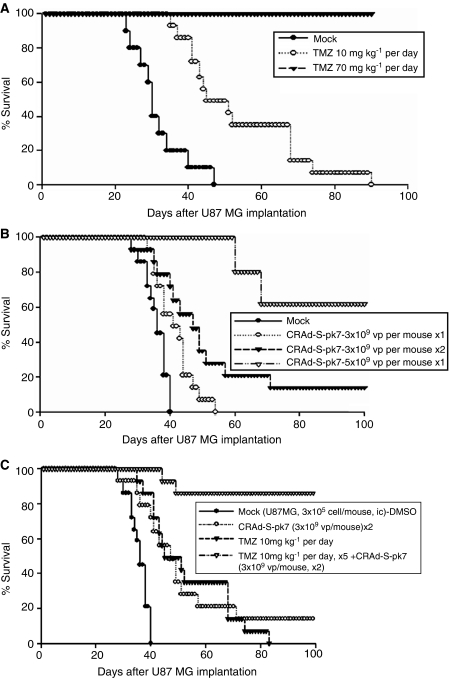
*In vivo* anti-tumour activity of CRAd-S-pk7 in combination with TMZ. (**A**) Five consecutive injections of TMZ at 70 mg kg^−1^ per day (▾) achieve 100% survival for 80 days after U87MG implantation, whereas five TMZ injections at 10 mg kg^−1^ per day (○) had significant increase in survival compared with mock treatment (•) (*P*<0.05). (**B**) Single intracranial (i.c.) injection of CRAd-S-pk7 at 5 × 10^9^ vp per mouse (▵) achieves significant increase in survival compared with mice that received two (○) or one (▾) injection of CRAd-S-pk7 at a dose of 3 × 10^9^ vp per mouse or mock control (•) (*P*<0.05) (**C**) Five consecutive injections of TMZ at 10 mg kg^−1^ per day followed by two CRAd-S-pk7 treatments each at 3 × 10^9^ vp per mouse (Δ) showed significant additive effect on mice survival compared with mock (▪) (*P*<0.02), double injections of CRAd-S-pk7 each at 3 × 10^9^ vp per mouse (▵) (*P*<0.02) or five consecutive injections of TMZ at 10 mg kg^−1^ per day (▾) (*P*<0.02).

The efficacy of TMZ and CRAd-S-pk7 combination was evaluated *in vivo* in mice with U87MG i.c. glioma xenografts. To employ a dose that resembles the partial therapeutic effect of TMZ seen in the clinical scenario (Stupp *et al*, 2005), different doses of TMZ were tested. On the basis of those studies, we chose a dose of 10 mg kg^−1^ per day for 5 days of i.p. TMZ to study the efficacy of TMZ and CRAd-S-pk7 combination, as this dose led to an increase in survival, but remained non-curative (Figure 4A) (log-rank test, *P*<0.05). Following the same rationale, we tested different doses of i.c. CRAd-S-pk7 injection to investigate which dose provides an increase in survival that could be further enhanced by the addition of co-adjuvant TMZ. We chose a CRAd-S-pk7 dose of 3 × 10^9^ vp per mouse × 2 injections for testing the therapeutic effects of TMZ and CRAd-S-pk7 combination, as it led to a partial efficacy that could be further enhanced (Figure 4B) (log-rank test *P*<0.05).

On the basis of the above findings, the combination of TMZ and CRAd-S-pk7 was tested for efficacy in terms of survival (Figure 4C). The combination of TMZ (10 mg kg^−1^ per day × 5) and two i.c. injections of CRAd-S-pk7 3 × 10^9^ vp per mouse led to a 90% of long-term survivors (>90 days) (LTS). In contrast, treatment with TMZ 10 mg kg^−1^ per day × 5 alone led to a median survival of 51 days (standard error (s.e.) 2.45) with 7% LTS, treatment with CRAd-S-pk7 3 × 10^9^ vp per mouse × 2 alone led to a median survival of 49 days (s.e. 2.0) with 14% LTS, and mock treated animals had a median survival of 37 days (s.e. 0.5) with no LTS (Figure 4C) (log-rank test, *P*<0.01). Consistent with the finding of an additive cytotoxic effect of TMZ and CRAd-S-pk7 *in vitro* experiments, this treatment combination led to an improved survival in mice bearing i.c. human glioma xenografts.

### TMZ and CRAd-S-pk7 lead to intra-tumoural expression of LC3 and cleaved Caspase-3 in mice bearing intracranial glioma xenografts

**Figure 5:**
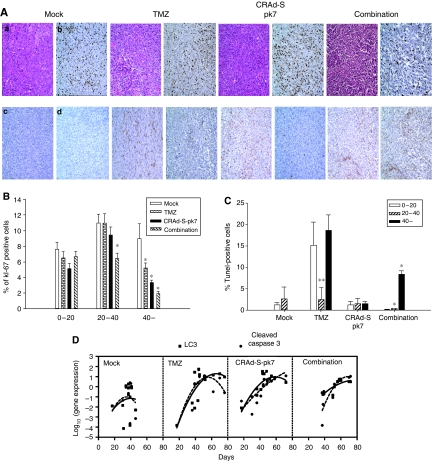
Histopathological analysis of U87MG glioma xenografts. Mice with i.c. xenografts were treated with different regimens and the brain tissue was harvested at specific time points. (**A**) Sections (at day 41 after tumour implantation) were stained with H&E (*a*) or with antibodies against Ki-67 (*b*), LC3 (*c*), or cleaved Caspase-3 (*d*). Percent averages of Ki-67 (**B**) or TUNEL (**C**) positive cells at different time points (days) after treatment with indicated course. ‘^*^’ represents *P*-value <0.05 compared with mock treatment. (**D**) The polynomial model was used to determine the relationship between the level of LC3 or cleaved Caspase-3 expressions and days after treatment. There was no difference between any of the treatment groups and only a marginal difference in the group shown additive effect (*P*=0.11). Solid lines represent a model fit for LC3, whereas the dashed line represents a model fit for cleaved Caspase-3.

The therapeutic effects of TMZ and CRAd-S-pk7 on i.c. glioma xenografts are additive and lead to a significant increase in survival. To evaluate the effect of our treatments, we performed an immunohistochemical analysis for Ki-67, LC3, TUNEL and cleaved Caspase-3. To do so, we selected a tumour area from each mouse specimen based on H&E staining viewed under × 20 magnification. The increase in survival seen in mice treated with TMZ and CRAd-S-pk7 combination was accompanied by a decrease in the expression of Ki-67, suggesting a decrease in proliferation of live glioma cells treated with this therapeutic combination (Figure 5A and B). To gain a better understanding of the mechanism of cell killing in the combination group *in vivo*, the expression of LC3, cleaved Caspase-3 and TUNEL staining was analysed semiquantitatively. Given the potential role of apoptosis in tumour death, we calculated the apoptotic index for each group at three different time points, 0–20, 20–40 and after 40+ days post treatments. As shown in Figure 5C, TMZ, CRAd-S-pk7 and their combination showed different patterns of TUNEL expression. Statistical analysis revealed that there was no significant difference between the percent of apoptotic cells measured at three different time points for mice receiving oncolytic virus injection. In contrast, mice receiving TMZ treatment followed by CRAd-S-pk7 injection showed elevated expression of TUNEL positive cells, especially after 40 days. However, even in the combination group, less than 10% of the cells were TUNEL positive, suggesting that although apoptosis plays a role, it is not an exclusive mechanism of cell death *in vivo*.

To further investigate the relationship between apoptosis and autophagy *in vivo*, we used nonlinear regressional analysis (Figure 5D). A two-order polynomial model for LC3 and cleaved Caspase-3 staining revealed direct increase in both markers over time, with no significant difference between any of the treatment groups and only a marginal difference with regard to cleaved Caspase-3 expression in the co-treatment group (*P*=0.11). These results suggest that both autophagy and apoptosis are responsible for the therapeutic efficacy observed with either therapy or combination treatment in an animal model *in vivo*.

## Discussion

In this study, we describe the therapeutic effect of an oncolytic adenoviral vector, CRAd-S-pk7, and TMZ, a clinically available alkylating agent, in the treatment of human gliomas. The additive cytotoxicity elicited by this treatment combination leads to a significant prolongation of survival in mice bearing i.c. human glioma xenografts.

The precise mechanism, by which CRAd-S-pk7 and TMZ potentiate each other, is not totally understood. We find that *in vitro*, autophagy is responsible for the cytotoxicity induced in response to the CRAd treatment, and this is particularly true for the combination group and for CRAd treatment alone. This finding is supported by a study performed by Jiang *et al* (2007), in which the authors first showed this mechanism of glioma cells killing by an oncolytic vector. In the TMZ treated group, there is no change in toxicity in response to BAF treatment, a late stage autophagy inhibitor (Kanzawa *et al*, 2003b), but we observed decreased toxicity in response to 3-MA, an early inhibitor of autophagy.

Recently, it was shown that an oncolytic adenovirus containing a mutation in the E1A region also exhibits non-classical apoptosis in an ovarian cancer model as evidenced by lack of Caspase-3 activation (Baird *et al*, 2008). This study supports our findings of non-dominant role of apoptosis in CRAd-mediated cell death *in vitro*. Along with apoptosis, we also evaluated other types of drug-mediated cell death. Cell cycle analysis along with Annexin V/7AAD staining and Western blot analysis revealed significant inhibition of cell progression in G1 (CRAd-S-pk7 treated) and G2/M (for TMZ treated cells) phase of the cell cycle. Presence of an active spindle checkpoint was elucidated by Western blots showing negligible Cdc20 levels and therefore, lack of MC as main mechanism behind the additive effect of TMZ and CRAd-S-pk7 therapy. Consistent with earlier studies performed in gliomas, in which the cytopathic effect elicited by a CRAd (Ito *et al*, 2006) or TMZ (Kanzawa *et al*, 2003a, 2004; Lefranc and Kiss, 2006) was attributed to autophagy, we have found that CRAd-S-pk7 and TMZ or their combination triggers autophagic cell death in U87MG glioma cells *in vitro*. However, our results are unique in the sense that we have performed an analysis of treated tumour *ex vivo*, and found that both autophagy, as well as apoptosis plays a role in therapeutic efficacy observed *in vivo* in experimental brain tumour models. To the best of our knowledge, this is the first time that analysis of cell death after oncolytic therapy has been reported *in vivo*. In fact, our results suggest, and we propose, that although autophagy may be the dominant form of cell death seen in the initial stages of therapy, animals that are long-term survivors attribute the therapeutic efficacy to both autophagy and apoptosis in the late stages of therapy.

The improved therapy exhibited by the TMZ and CRAd-S-pk7 cocktail adds to the rationale for testing CRAd-S-pk7 in the clinical scenario. The heterogeneous cellular makeup of gliomas, especially the CD133+ stem cell content, makes it difficult for a single therapy to be adequate in and of itself. The prevailing standard of care for patients bearing these tumours includes administration of TMZ, which although leads to prolongation of meaningful survival for patients, is far from optimal. The combination of TMZ and oncolytic virus inducing an additive effect against glioma cancer stem cells is an attractive option to consider. In fact, Jiang *et al* have earlier shown the therapeutic potential of Delta-24-RGD in brain tumour stem cells (Jiang *et al*, 2007). Our group has also recently shown that combination of low-dose radiation along with CRAd-S-pk7 successfully targets CD133+ glioma stem cells (Nandi *et al*, 2008). Another interesting application of oncolytic virotherapy was proposed by (Alonso *et al*, 2007), who first suggested using the virus to overcome resistance of tumour cells to TMZ. This is an important aspect of tumour therapy and underlines the importance of tumour co-treatment with oncolytic virus and TMZ. The ideal case, in which a novel treatment for GBM could be introduced at the bedside, is whether there is some evidence of efficacy in the adjuvant setting. We believe that this study supports the application of CRAd-S-pk7 oncolytic-based therapy along with TMZ in a clinical study.

## Figures and Tables

**Table 1:** Distribution of cells in cell cycle

**Type of treatment**	**Days after treatment**	**%Sub-G1**	**%G1**	**%S**	**%G2/M**	**%Polyploidy**
Mock	1	1.63	49.15	9.68	22.35	16.3
	3	2.32	61.95	6.01	20.1	8.98
	5	4.2	63.2	5.05	13.65	13.25
	10	1.68	66.6	4.51	15.5	9.89
TMZ	1	3.26	47.95	11.55	22.6	14.55
	3	1.4	20.1	12.6	50.7	14.15
	5	1.63	6	4.27	61.15	27.85
	10	1.18	9.2	6.19	50.1	32.4
CRAd-S-pk7	1	2.07	49	12.45	22.65	14.5
	3	3.57	65.55	7.36	17.6	6.62
	5	4.58	60.3	3.3	15.8	13.35
	10	4.12	23.45	15.3	23.85	30.85
Combination	1	2.92	45.6	11.45	24.1	16.35
	3	2.21	33.5	10.6	39.7	12.3
	5	3.31	9.065	6.1	53.9	29.45
	10	2.35	11.65	7.2	38.4	40.5

Abbreviation: TMZ=temozolomide.
